# Locals

**Published:** 1879-02-20

**Authors:** 


### *LOCALS.*

PHOSPHORIZED COD LIVER OIL.—See advertisement of the above combination from the reliable and enterprising house of BILLINGS CLAPP & Co., Boston.

ALLAIRE WOODWARD & Co.—See their new advertisement in the present no. of our journal. This is a safe house, vigorous and enterprising business men.

WM. R. WARNER & Co.. of Philadelphia, have a new advertisement in present number of our journal. Large establishment, extensive manufacturers and high reputation as business men.

WESLYAN ADVOCATE.—This excellent paper is published in Macon, Ga., by J. W. BURKE & Co. See advertisement.

